# *In Vitro* to *In Vivo* Extrapolation Linked to Physiologically Based Pharmacokinetic Models for Assessing the Brain Drug Disposition

**DOI:** 10.1208/s12248-021-00675-w

**Published:** 2022-01-13

**Authors:** Yukiko Murata, Sibylle Neuhoff, Amin Rostami-Hodjegan, Hiroyuki Takita, Zubida M. Al-Majdoub, Kayode Ogungbenro

**Affiliations:** 1 Centre for Applied Pharmacokinetic Research, Division of Pharmacy and Optometry, University of Manchester, Manchester, M13 9PT UK; 2 Sohyaku.Innovative Research Division, Mitsubishi Tanabe Pharma Corporation, 1000, Kamoshida-cho, Aoba-ku, Yokohama, Kanagawa 227-0033 Japan; 3 Certara UK Ltd, Simcyp Division, 1 Concourse Way, Level 2-Acero, Sheffield, S1 2BJ UK; 4 Development Planning, Clinical Development Center, Asahi Kasei Pharma Corporation, Hibiya Mitsui Tower, 1-1-2 Yurakucho, Chiyoda-ku, Tokyo, 100-0006 Japan

**Keywords:** central nervous system, blood-brain barrier, drug transporters, lipophilicity, tissue concentrations

## Abstract

Drug development for the central nervous system (CNS) is a complex endeavour with low success rates, as the structural complexity of the brain and specifically the blood-brain barrier (BBB) poses tremendous challenges. Several *in vitro* brain systems have been evaluated, but the ultimate use of these data in terms of translation to human brain concentration profiles remains to be fully developed. Thus, linking up *in vitro-to-in vivo* extrapolation (IVIVE) strategies to physiologically based pharmacokinetic (PBPK) models of brain is a useful effort that allows better prediction of drug concentrations in CNS components. Such models may overcome some known aspects of inter-species differences in CNS drug disposition. Required physiological (i.e. systems) parameters in the model are derived from quantitative values in each organ. However, due to the inability to directly measure brain concentrations in humans, compound-specific (drug) parameters are often obtained from *in silico* or *in vitro* studies. Such data are translated through IVIVE which could be also applied to preclinical *in vivo* observations. In such exercises, the limitations of the assays and inter-species differences should be adequately understood in order to verify these predictions with the observed concentration data. This report summarizes the state of IVIVE-PBPK-linked models and discusses shortcomings and areas of further research for better prediction of CNS drug disposition.

**Figure:**
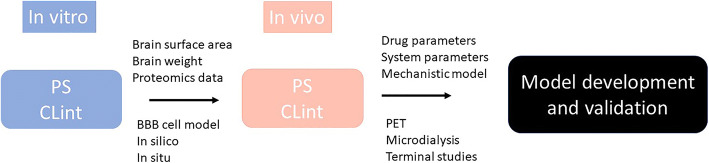
Graphical abstract

Graphical abstract

## INTRODUCTION

**Fig. 1:**
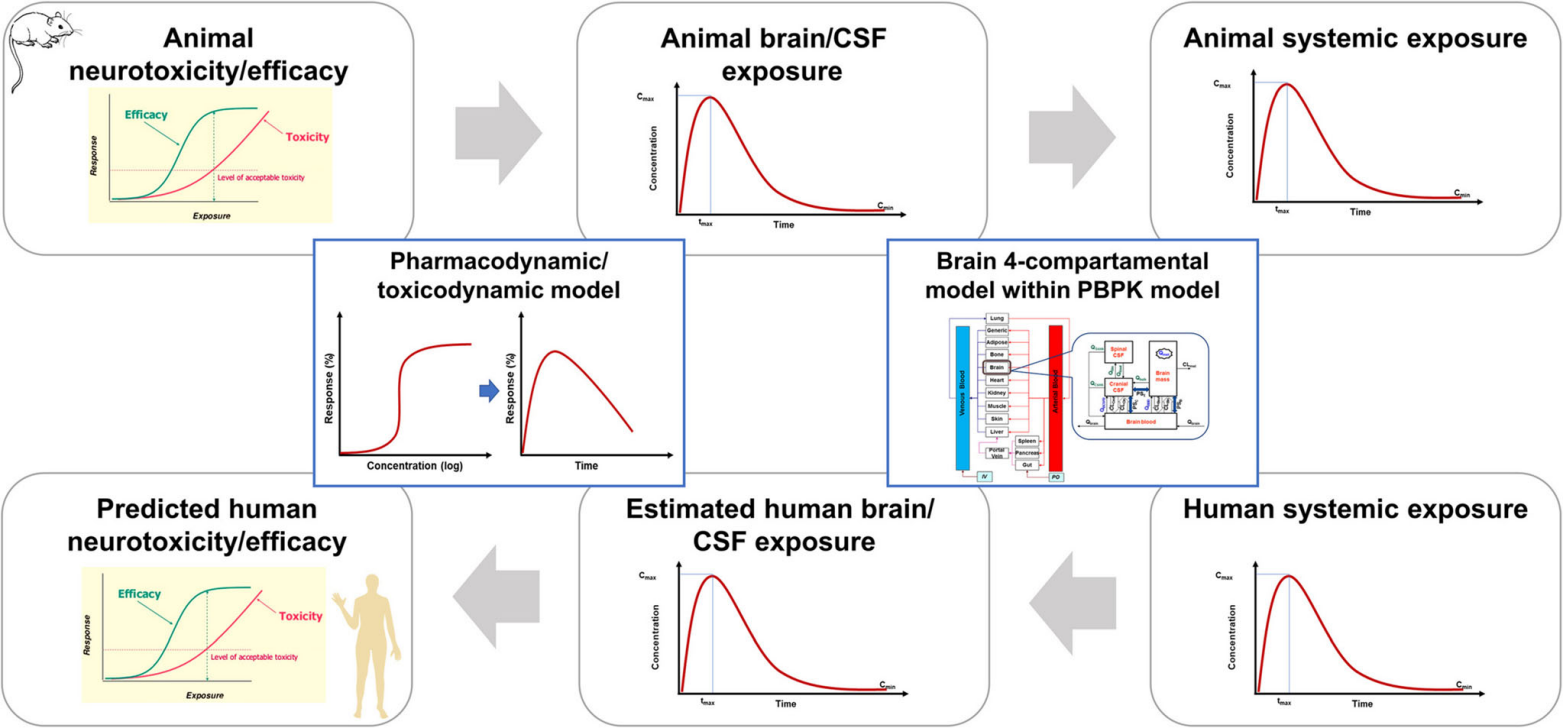
Prediction of pharmacological efficacy and neurotoxicity using brain physiologically based pharmacokinetic (PBPK/PD) modelling in the translation from pre-clinical efficacy/neurotoxicity studies. Figure is adapted from Fig. 1 of Al Feteisi *et al*., (3)

Drug development is costly and improving the productivity requires more rationale progression of compounds with high viability to advanced phases of development. It is estimated that 90% of industry R&D expenditure goes into molecules that never reach the market (1). Hence, making the right decision on what to progress to late-stage clinical trials is essential. The rate of failure is similar for most therapeutic areas, but particularly neuroscience has been deemed a more difficult area with lower rate of success (1,2). Whilst a large part of this relates to lack of good experimental models mimicking relevant mechanisms of the disease, the difficulties associated with the location of the drug effect, namely central nervous system (CNS), cannot be dismissed. Unlike many other organs in which drugs in the systemic circulation readily diffuse, there is a blood-brain barrier (BBB) and a blood-CSF barrier (BCSFB) in the brain that control drug diffusion within and outside the brain. Particularly, BBB has many features that makes establishing relationship between the drug concentrations in systemic circulation and in CNS more challenging. The BBB tightly regulates the exchange of molecules with systemic circulation via its micro-structure and transport proteins. Although, pre-clinical animal models (mainly rodents) are used in neuroscience and neurotoxicity research, the abundance and nature of the transporters in rodents vary from humans (3,4). Consideration of such inter-species differences are important in the translation of observations in animals to expected outcomes in humans. A physiologically-based pharmacokinetic (PBPK) modelling framework is suggested that requires quantitative knowledge of transport (passive and active) in each species, the experimental affinity of the molecules to various transporters, and ‘local exposure’ in animals that be projected to expected toxicological or pharmacological effects in humans (3) (Fig. 1).

PBPK models based on human physiology allow prediction of drug concentrations in target tissues, which has been well documented and have become a critical tool in nonclinical and clinical study design and regulatory review. At the same time, CNS PBPK models have been reported based on various types of model structures and parameter acquisition methods. Physiological parameters in the model are generally derived from intrinsic quantitative values in each organ, while compound-specific parameters are derived based on *in silico* or *in vitro* experiments and translated via *in vitro-to-in vivo* extrapolation (IVIVE), and may include preclinical *in vivo* results too (5). In this review, we describe the structure of the CNS, factors that determine the central distribution of drugs, and methods for experimental evaluation. In addition, differences among published brain PBPK models are highlighted and compared, to provide a perspective for CNS evaluation using the brain IVIVE-PBPK model.

## STRUCTURE OF THE CNS

CNS consists of the brain and spinal cord, surrounded by meninges and skull (6). The spaces between the arachnoid membrane and spinal cord or brain, including the ventricles, are filled with medium called spinal or cranial cerebrospinal fluid (CSF), respectively. Interstitial fluid (ISF) occupies intercellular space of the brain (20% of the total brain volume of around 1250 mL in humans (7,8)), mediating the exchange of drugs between brain cells and CSF. Microvessels, which are intricately branched from cerebral arteries, carry oxygen and nutrients from blood to the brain, while microvessel endothelial cells prevent the penetration of harmful substances into the brain by forming tight junctions, adherens junctions and the BBB. Endothelial cells of the choroid plexus and arachnoid membrane work as another barrier between blood and CSF (blood-cerebrospinal fluid barrier, BCSFB), with a significant smaller surface area than the BBB (~50% of BBB (9)). Since predominantly protein-unbound unionized drugs penetrate these barriers, the distribution of drugs in the brain is determined by factors like the pH (pH; 7.3(10)) or protein content (≈ 0.2 g/L (8,11)) of CSF/ISF and plasma. In addition to the limitaion in passive permeation, active (blood orientated) effflux carriers located in the BBB and BCSFB work as second defense system for the brain by excreting drugs out of the brain. After reaching the brain, drugs distribute throughout the CNS by the flow of CSF (0.2–0.4 mL/min in human (8,12)). Produced in the choroid plexus, CSF ascends to the superficial subarachnoid space or down the spinal space, and is finally absorbed by various routes such as arachnoid granules or lymphatic vessels. The total CSF (140 mL in human) is replaced 2-4 times per day (6,8). To explain drug disposition in the brain, it is necessary to understand not only properties of the drug itself, but also these physiology of CNS; physiological parameters such as volumes of micro-compartments or flow rates of mediums in human and aminals as summarised previously (10,12). For drugs targeting CNS diseases, it is also important to consider the influence of the diseases on the physiology of the brain; CNS diseases such as stroke, brain tumor, and meningitis, as well as aging, may change barrier function (BBB, BCSFB) or composition and/or flow rate of the CSF and/or ISF. leading to altered drug disposition in the brain (6,7).

## TECHNIQUES TO ESTIMATE BRAIN DISTRIBUTION OF CNS AND TRANSPORT ACROSS THE BBB

Several approaches have been developed to evaluate membrane permeability and pharmacokinetic (PK) in the brain, including *in vivo*, *in vitro*, *ex vivo,* and *in silico* methods (Table I). This part summarizes the methods useful for understanding brain distribution and discusses the approaches currently predominantly used for CNS exposure prediction.

**Table I:** Methods to Estimate Brain Distribution and Transport of Drugs

Method	Measured/Estimated Parameter	Advantages	Disadvantages
*In vivo* methods			
Intravenous injection/ brain sampling	Influx; influx & efflux	Most physiological approach Highest sensitivity Low technical difficulty	Not simple to discriminate unidirectional uptake from bidirectional transfer.
Brain uptake index (BUI) BUI is defined as the relative percentage of uptake of the test compound and reference compound injected into the brain	Influx	Fast procedure Moderate technical difficulty Permits wide range of modifications of injectate composition Artifacts by metabolism largely excluded	Relatively insensitive (compared with intravenous injection and brain perfusion)
Brain efflux index (BEI) BEI is defined as the relative percentage of drug efflux from the ipsilateral cerebrum.	Efflux	Moderate technical difficulty Permits wide range of modifications of injectate composition Artifacts by metabolism largely excluded	Technically more difficult and invasive than intravenous experiments.
Brain perfusion	Influx	Higher sensitivity compared with BUI Permits modification of both perfusate composition and flow rates Artifacts by peripheral metabolism are excluded	Technically more difficult than intravenous experiments and BUI
Quantitative autoradiography	Influx	Excellent spatial resolution	Time-consuming evaluation No proof of integrity of tracer
External registration: MRI, SPECT, PET	Influx/efflux; K_p,brain_	Noninvasive and applicable in humans Allows time course measurements in individual subjects	Expensive equipment (MRI, PET) and tracers (PET) Limited sensitivity (MRI) and availability of labeled tracers (MRI, PET) Poor spatial resolution for small animals (SPECT)
Microdialysis	Influx/efflux; V_u,brain,MD_	Allows time course measurements in individual subjects Samples well suited for subsequent analytical procedures	Technically involved i*n vivo* probe calibration required for valid quantitative evaluation Local damage to BBB integrity Nonspecific binding is a challenge
CSF sampling	Influx/efflux	Readily accessible for sampling; Applicable to humans	Reflects permeability of BCSFB and CSF fluid dynamics rather than BBB
*In vitro* methods			
Brain slices	Uptake;V_u,brain_	High-throughput method has been developed; Active transport systems, pH gradients, and cell-cell interactions are conserved. The method provides information relevant to nonspecific binding to brain tissue, lysosomal trapping, and active uptake into the cells. The method is suitable for estimation of target-site pharmacokinetics in the early drug discovery process and fundamental pharmacological studies.	Technically more difficult than intravenous experiments and BUI. Technical skill is required
Brain homogenate	fu,brain; V_u,brain_	Measures the intracellular binding by equilibrium dialysis of diluted brain homogenates and allows estimation of Vu, cells and the buffer-to-homogenate concentration ratio.	Active transport systems, pH gradients, and cell-cell interactions are not conserved Dilution corrections are required
Fresh isolated brain microvessels	Binding, uptake, efflux	Representing the *in vivo* expression of transporters and efflux systems at the BBB	Transcellular passage cannot be measured Microvessel preparation is a challenge
EC membrane vesicles	Carrier-mediated transport	Allows distinction of luminal *versus* abluminal transport activity	Large amounts of source material required laborious preparation
Endothelial cell culture			
Primary cultures, cell lines	Receptor binding Uptake Luminal to abluminal transfer (and opposite direction)	Permeability screening experiments (feasible with primary EC from bovine/porcine sources) Effect of culture conditions on BBB transport properties may be studied (e.g., astroglial factors, serum effects, inflammatory stimuli, hypoxia/aglycemia)	No system yet able to represent *in vivo* condition with respect to barrier tightness and BBB specific transporter expression Multitude of models makes comparison of results between studies difficult
Epithelial cell culture			
Cell lines (such as Caco-2. MDCK, LLC-PK_1_ and their transfections)	Uptake Efflux Bidirectional transport	Permeability screening experiments Transporter kinetics Simple systems that are routinely established in industry	Not all transporters will function the same in the epithelial system *versus* the BBB The passive transcellular and paracellular pathways are different in epithelial and endothelial cells
Artificial membranes			
Parallel artificial membrane permeability assay (PAMPA) Several PAMPA for the brain have been developed. It is a method which determines the permeability of substances from a donor compartment, through a lipid-infused artificial membrane into an acceptor compartment.	Passive permeability	High-throughput method Low cost	No information on active transport or metabolism There are several different PAMPA with different degree of success
*In silico* models			
QSAR	CNS active (+/−) Log BB Log PS	Screening of large compound libraries (depending on model selection and computational resources) Screening of virtual libraries	Many current models based on data, which may not represent BBB permeability as such (log BB; CNS activity) Still very limited data bases for BBB transport (log PS models)

### Positron Emission Tomography (PET)

PET is a non-invasive method for measuring concentrations of positron emitting radioisotopes. Imaging by PET allows the Kp,brain (total concentration ratio of the brain to plasma) of the radiolabelled drugs to be determined in humans (13–15). The method cannot distinguish between free and bound compounds and metabolites from parent compounds. In addition, since it targets the brain, it must be a highly lipophilic compound, but non-specific adsorption may occur, also there are limitations such as the short half-life of isotopes used in PET. Consequently, PET data are only available for a limited number of CNS drugs (16).

### *In Vivo* Microdialysis

The unbound extracellular concentration and time profiles in each CNS compartment provide important information for drug distribution, but the only way to know this information *in vivo* is by microdialysis. The unbound volume of distribution in the brain (V_u,brain_) determined by microdialysis (V_u,brain,MD_) is calculated by dividing the amount of drug in the brain (A_brain)_ measured by conventional brain tissue sampling by the unbound drug concentration in the brain ISF (C_u,brainISF)_ measured by microdialysis in the same animal (Eq. 1)(17).

1 $$ {V}_{u, brain, MD}=\frac{A_{brain}}{C_{u, brain, ISF}} $$

### *In Vitro* Brain Slice Methods

The brain slice method was originally developed by Kakee *et al*.,(18) and further refined by Friden (17). The high-throughput brain slice method is a precise and robust technique for estimating the overall uptake of drugs into brain tissue through determination of the unbound volume of distribution in the brain (V_u,brain_; ml·g brain^-1^) (19). By measuring concentrations in brain slices and buffers at steady state, unbound V_u,brain_ can be calculated without the need for microdialysis. The brain slice method is more physiologically based than the brain homogenate method with respect to the assessment of drug distribution in the brain since active transport systems, pH gradients, and cell-cell interactions are conserved. The method provides information relevant to nonspecific binding to brain tissue, lysosomal trapping, and active uptake into the cells. For these reasons, the brain slice method is suitable for estimation of target-site PK in the early drug discovery process and fundamental pharmacological studies.

### *In Vitro* Brain Homogenate Assay

The brain homogenate binding method measures the intracellular binding by equilibrium dialysis of diluted brain homogenates and allows estimation of V_u,brain_. The fraction of unbound drug in diluted brain homogenate, f_u,hD_, i.e. the buffer-to-homogenate concentration ratio, is used to calculate f_u,brain_, while also taking into account the dilution (D) associated with homogenate preparation (Eq. 2)(20). The inverse of fu,brain (Vu,brain(h)) is used to express the quantity on the Vu,brain scale (Eq. 3)(17).

2 $$ {\boldsymbol{f}}_{\boldsymbol{u},\boldsymbol{brain}}=\frac{\mathbf{1}}{\mathbf{1}+\boldsymbol{D}\left({\frac{\mathbf{1}}{\boldsymbol{f}}}_{\boldsymbol{u},\boldsymbol{hD}}-\mathbf{1}\right)} $$

3 $$ {\boldsymbol{V}}_{\boldsymbol{u},\boldsymbol{brain}\left(\boldsymbol{h}\right)}=\frac{\mathbf{1}}{{\boldsymbol{f}}_{\boldsymbol{u},\boldsymbol{brain}}} $$

### *In Situ* Brain Perfusion

A widely used method to measure the permeability of the BBB *in vivo* is the *in situ* brain perfusion technique. The *in situ* perfusion method was originally developed by Takasato for the rat (21). However, it has been expended to be used in mice, guinea pigs and rabbits. This method generates a permeability surface area-product (PS) in ml/s/g (brain weight). PS could be converted into *in vivo* brain permeability values (Pe *in vivo* in cm/s) by dividing PS by an estimated value of the surface area (S) of perfused capillaries equal to 150 cm^2^/g of brain in rat (22).

### BBB Cell Models

#### Cell Culture Models to Estimate Transporter Kinetics

Standard permeability systems in the industry are Caco-2, MDCK and LLC-PK_1_ cell systems. However, there are several sources of these three cell systems (23), hence a comparison between these systems is often challenging. Users of this models often rely on in-house experience and correlations. The systems are well-established and regularly used for the estimates of intestinal permeability in general research and for regulatory submissions. It is therefore not surprising that these systems, although epithelial cell based, are tested to be used to give not only transporter kinetic data, but also permeability estimates for the BBB (24). Transporter kinetic obtained from such cell systems (mostly MDCK-MDR1, LLC-PK_1_-MDR1, MDCK-BCRP, and Caco-2), specifically when they are only driven by ATP-dependent transporter like MDR1 and BCRP, can be used, since the functionality of these transporters in the brain as matrix is unlikely different to the functionality in the *in vitro* cell system (as long as the driving force, i.e. ATP is supplied) (25,26). The passive permeability, however, may be different and the user should be aware of the assumptions made, when using these *in vitro* systems to estimate the passive permeability across the BBB.

### Brain Uptake Index and Brain Efflux Index Methods

The brain uptake index (BUI) is defined as the relative percentage of uptake of the test compound and reference compound injected and easily penetrate the brain but not for poorly penetrating compounds (27). The Brain Efflux Index (BEI) is defined as the relative percentage of drug efflux from the ipsilateral cerebrum (18). These methods allow investigation of carrier-mediated BBB transport.

### Parallel Artificial Membrane Permeability Assay (PAMPA)

Several PAMPA for the brain have been developed (28,29). It is a method which determines the permeability of substances from a donor compartment, through a lipid-infused artificial membrane into an acceptor compartment. PAMPA-BBB cannot account for transporter effects and is solely representing an artificial membrane that mimics the BBB to estimate the passive permeation at that barrier.

### *In Silico* Quantitative Structure Activity Relationship (QSAR) Models

Numerous QSAR models have been developed for predicting the log BB (the logarithm value of brain to blood concentration ratio) (30–32) and the log PS (the logarithm value of permeability surface area product) (33–35). As data in humans are sparse these QSAR models are generally established based upon non-human, generally rat data. Consequently, a correction is required when extrapolating these predictions to humans.

## PBPK MODELS FOR BRAIN

Broadly, in inreasining order of complexity models in PK can be classified into empirical, semi-mechanistic and mechanistic (PBPK) models (36). This classification is also based on the amount and quality of data required and the intended goal of the analysis. An empirical model development is based on a broad understanding of human physiology, the body is assumed to be composed of 1, 2 or 3 homogenous compartments. This approach is mostly top down, data driven and limited in usage. The semi-mechanistic model developmement incorporates mechanistic understaning of certain components of the body, which allows the developer to focus on specific area of the body and also helps to reduce complexity. Some parameters of these models are supported by anatomy and physiology and *in vitro/in silico* experiments, the rest are estimated from *in vivo* data. The PBPK modelling on the other hand is based on mechanistic understanding of the physiology of the body; various tissues and organs are represented as compartments, volumes are actual physiological volumes and connections are by physiological flows. PBPK modelling is often based on a bottom up approach, where data from *in vitro* and *in silico* experiments are intergrated with other physiological data during development. Consequently, PBPK models are generally very rich in information content and can be used in wider scenario, especially for extrapolation during drug development (37). The advancement of methodologies for integrating *in vitro* and *in silico* data under IVIVE paradigm together with PBPK models in the last 20 years has increased the usefulness and application of PBPK models (5).

Based on the structure of central nervous system presented above, published models in the literature for desciption/prediction of brain exposure of drugs in animals and humans will be discussed in this review. Table II gives a summary of literature models these categories: empirical, semi-mechanistic or PBPK (IVIVE) models. The table also states the model properties such as the specie the model was developed for, data available during development, the stucture of the model (compartments for brain and CSF), model parameter for active transport and how this parameter was derived, how the model for the rest of the body was handled, i.e. disposition and the source of data. In most cases, the models have been developed in rats, due to the need to obtain samples during the experiment from the brain and CSF in addition to blood for accurate characterisation of dispostion in the CNS. This includes microdialysis experiments, which allows direct quantification of endogenous and exogenous substances in different regions of the central nervous system such as the CSF regions, intracellular and extracelluar areas. Also, terminal brains and CSF concentrations have been used as sources of data for model development in animals. For the CSF model developed based on human data, human CSF and brain samples in disease population in addition to plasma data have been used (38,39).

**Table II:** Summary of Literature Brain Models

No	Species	Brain model structure				Passive transport	Transporter mediated transport	Volume of each compartment	Rat PK data	Human PK data	Compound	Platform	Refs
		Brain blood/plasma	ISF (ECF)	ICF	CSF								
1	rat	x	x	-	-	Parameter estimation (*in vivo*)	Parameter estimation (*in vivo*)	Parameter estimation (*in vivo*)	MD	-	Morphine	NONMEM vV	(40)
2	rat	x	x(2 compartments)		-	Parameter estimation (*in vivo*)	Parameter estimation (*in vivo*)	Parameter estimation (*in vivo*)	MD	-	Fluvoxamine	NONMEM vV	(81)
3	rat	x	x	-	x	Parameter estimation (*in vivo*)	Parameter estimation (*in vivo*)	Parameter estimation (*in vivo*)/Physiological values	MD	-	Quinolones	MULTI	(41)
4	rat	x	x	x	x	Parameter estimation (*in vivo*)	Parameter estimation (*in vivo*)	Brain slice/Physiological values	*in vivo* PK	-	3'-Azido-3'-deoxythymidine, 2',3'-Dideoxyinosine	MULTI	(42)
5	rat	x	x	x	x	Parameter estimation (*in vivo*)/*In Situ* brain perfusion	Parameter estimation (*in vivo*)	Brain slice	MD	-	Morphine, morphine-6-h-d-glucuronide	WinNonlin v4.1	(43)
6	rat	x(extravascular, intravascular)	x		-	Parameter estimation (*in vivo*)/*In Situ* brain perfusion	-	Parameter estimation (*in vivo*)/brain homogenate	*in vivo* PK	-	Caffeine, fluoxetine, propranolol, theobromine, theophylline, NFPS, CP-141938	WinNonlin v3.2	(44)
7	rat, (human : simulation only)	x	x	x (brain cells)	x	Parameter estimation (*in vivo*)	Parameter estimation (*in vivo*)	Physiological values	MD	-	Duloxetine, Atomoxetine	NONMEM vV	(45)
8	rat,human	x	x	(x)	x(LV,TFV,CM,SAS)	Parameter estimation (*in vivo*)	-	Physiological values	MD	x	Paracetamol	NONMEM v6.2	(46)
9	rat	x	x	x (brain deep)	x(LV,TFV,CM,SAS)	Parameter estimation (*in vivo*)	Parameter estimation (*in vivo*+inhibitor)	Physiological values	MD	-	Quinidine	NONMEM v6.2	(47)
10	rat,dog, human child, human adult	x	x	-	x(LV,TFV,CM,SAS)	Parameter estimation (*in vivo*)	Parameter estimation (*in vivo*+inhibitor)	Physiological values	microdialysis	x	Methotrexate	NONMEM v6.2	(48)
11	rat,human	x	x	x	x (LV,TFV,CM,SAS)	Parameter estimation (*in vivo*)	Parameter estimation (*in vivo*)	Physiological values	MD	x	Paracetamol,Atenolol,Methotrexate,Morphine,Quinidine,Remoxipride,Paliperidone,Phenytoin,Risperidone	NONMEM v7.3	(49)
12	rat	x(microvascular)	x	x/lysosome	x(LV,TFV,CM,SAS)	*in silico*	*in silico*	Physiological values	MD	-	Paracetamol, Atenolol, Methotrexate, Morphine, Quinidine, Remoxipride, Paliperidone, Phenytoin, Risperidone, Raclopride	NONMEM v7.3	(50)
13	human	x(microvascular)	x	x/lysosome	x(LV,TFV,CM,SAS)	*in silico*	translational method from rat	Physiological values	-	x	Paracetamol,Oxycodone,Morphine,Phenytoin	NONMEM v7.3	(51)
14	mouse	x	x		-	IVIVE(Caco2 Papp)	IVIVE(Caco2 Papp)	Physiological values	-	-	Domperidone	S-Plus	(55)
15	rat, (human : simulation only)	x(vascular)	x		-	IVIVE(Caco2 Papp)	IVIVE(Caco2 Papp,TR-BBB13,RAF)	Physiological values	MD	x(plasma)	Morphine,Oxycodone	acslX	(57)
16	rat	x	x	x	x	Parameter estimation (*in vivo*)/IVIVE(Caco2 Papp)	Parameter estimation (*in vivo*)/IVIVE(Caco2 Papp)	Physiological values	MD	-	Paracetamol,atomoxetine,S 18986	WinNonlin v6.3	(58)
17	rat,human	x	x	x	-	Parameter estimation (*in vivo*)/IVIVE(Caco2 Papp)	Parameter estimation (*in vivo*)/IVIVE (Caco2 Papp)	Physiological values	MD	x(plasma)	Anonymous	WinNonlin v6.3	(82)
18	mouse,rat	x(vascular, intravascular)	x	-	x (CSF, choroid plexus )	IVIVE(LLC-PK1 Papp)	IVIVE(ER, REF)	Physiological values	MD	-	norfloxacin	Matlab v8.1	(59)
19	human	x	x		x(cranial,spinal)	*in situ* brain perfusion	Parameter estimation (*in vivo*)	Physiological values	-	x	Paracetamol,Phenytoin	Simcyp Simulator v14	(38)
20	human	x	x	-	x(cranial,spinal)	IVIVE(MDCKIIPapp)	IVIVE(MDCKIIPapp, ER,HEK293,REF)	Physiological values	-	x(Glioblastoma Patients)	Anonymous	Simcyp Simulator v16	(39)
21	rat,human	x(vascular, extravascular)	x(striatum/cortex)	-	-	IVIVE(MDCKIIPapp)	IVIVE(MDCKIIPapp, ER, REF)	Physiological values	-	x	Clozapine,Haloperidol,Olanzapine,Paliperidone, Quetiapine,Risperidone	NONMEM vVI	(71)
22	rat,human	x	x(hippocampus/frontal cortex)	x(rest of brain tissue)	x	IVIVE(Papp)	IVIVE(ER,REF)	Physiological values	MD	x	Phenytoin,Carbamazepine,Morphine	Matlab v9.1	(60)
23	human child, human adult	x	x		x(cranial, spinal)	*in situ* brain perfusion/IVIVE (*in vitro* permeability)	-	Physiological values	-	x	Paracetamol, Ibuprofen,Flurbiprofen,Naproxen, Meropenem	R v1.1.442	(61)

*ECF* brain extracellular fluid, *ICF* brain intracellular fluid, *REF* relative transporter expression factor, *ER* efflux ratio, *LV* lateral ventricle, *TFV* the third and fourth ventricle, *CM* the cisterna magna, *SAS* the subarachnoid space, *MD* microdialysis

### Empirical Models

These models are generally obtained by fitting experimentally obtained *in vivo* plasma or blood, brain and CSF data in animals to empirically determined number of compartments. Physiological parameters such as tissue volumes or flows are not used or fixed, and these model have limited use since extrapolation especially between species have to be done with caution. An example of this type of model is the 4-compratment model developed for characterisation of the PK of morphine in male rats following short intravenous infusions, with and without continuous intravenous infusion of a MDR1 inhibitor (GF120918) (40). Total blood and unbound extracellular concentrations of morphine obtained by intracerebral microdialysis were used for the modelling. The model also included a 3-compartments for blood PK, which was not influenced by the MDR1 inhibitor and a 1-compartment model for unbound extracellular fluid (ECF) disposition obtained by microdialysis. The model included a parameters for passive diffusion and active saturable efflux and also nonlinear dose dependent distribution into the brain was captured.

### Semi-mechanistic Models

These models use knowledge of anatomy and physiology of the central nervous system to determine the structure and number of compartments for the model. Parameters of these models are either fixed to physiological values or estimated from the available *in vivo* data. This middle out approach combines prior information about the anatomy and physiology of the system with the information available in the *in vivo* data, this allows physiological interpretation of estimated parameters of the model. Ooie *et al*. (41) characterised the CNS distribution of quinolone antibiotics in rats using blood, CSF and whole brain terminal samples. With other parameters fixed to physiological values, permeability clearances across the BBB and BCSFB were estimated and used to provide evidence for efflux of quinolones across the BBB. Takasawa *et al*. (42) used the same model as Ooie *et al*. (41), to characterise the distribution of 3’-azido-3’-deoxythymidine and 2’,3’-dideoxyinosine in brain tissue and CSF. Bourasset *et al*., (43) developed a capacity-limited transport model for morphine and its metabolite, morphine-6-β-D-glucuronide using blood, brain (intra and extracellular samplind) and CSF concentrations in rats. In this study, the *in vivo* data was obtained from a microdialysis experiment and some model parameters were informed by prior data from *in situ* experiments and physiology. The remaining parameters were estimated by fitting the model to the observed data. Liu *et al*., (44) developed a hybrid brain PBPK model for seven compounds in rats using plasma and *in situ* brain data, with the assumption that there is no significant contribution to the disposition in the brain of the compounds under investigation by any transporter. Physiological parameters such as the volume and blood flow of rat brain were obtained from physiological data and other parameters for brain disposition were estimated from the *in vivo* data. Also, parameters derived from this model were correlated with corresponding parameters derived from *in situ* brain perfusion and equilibrum dialysis using brain homogenate. It was shown that for reaching a rapid brain equilibrium a high BBB permeability and a low brain tissue binding is required. Kielbasa *et al*., (45) developed a semi-mechanistic model for disposition of atomoxetine and duloxetine in rats using plasma, extracellular brain obtained by microdialysis, terminal CSF and whole brain concentrations following intravenous loading and infusion dosing. The model development was supported by physiological brain parameters obtained from rats (volumes), and *in vitro* binding (plasma and brain) parameters, while other brain drug disposition parameters were estimated. The model was also used to translate brain disposition from rats to humans by allometry. Westerhout *et al*., (46), developed a multi-compartment model (including five brain and CSF) for paracetamol in rats using data obtained by serial sampling of blood and microdialysis probes at different regions of CSF and brain. The main focus of the study was to quantify regional drug diffusion and fluid flow processes in the brain, and for this a no transporter substrate compound was used. In the model, the volumes of the brain and CSF compartments as well as brain and CSF flow in rats were fixed to physioloigcal values; however, clearace parameters, which were used to desribe the exchange between the compartments were estimated. The model was further used to predict human brain exposure using available human CSF concentration data from the subarchnoid space, and for this human parameter values were substituted for rat values in the model. This model was subsequently extended to include parameters for transporter function and was applied to other compounds and species (47–49). Also, Yamamoto *et al*., (50,51) extended the model further to include compartments for BBB and BCSFB. A subcellular compartment in the brain was introduced for lysosomes and pH-dependent drug partitioning was also introduced. Model parameterisation were also modified and distinction between system- and drug-specific parameters were introduced to improve the the performace of the models, especially the translation ability. This model which is now called LeiCNS-PK3.0 has been further updated to address other issues such as ionization, brain tissue non-sepcific binding and passive paracellular transport (10,52). Monine *et al*., (53) developed a multicompartment CNS model based on anatomy, this was used to describe the PK of antisense oligonucleotides in non-human primate following intrathecal administration to bypass BBB using physiological and drug specefic parameters estimated from *in vivo* data. Vendel *et al.,* (54) developed a mechanistic 3D model to explain local drug concentration within rat brain. The model represents the brain as a 3D cube unit; where the brain ECF is surrounded by capillaries. Drug transport through BBB was described by passive (paracellular and transcellular) and active transport processes, binding kinetics was also accounted for in the model. System and drug specific parameters for cappillaries (distance, radius, flow velocity), brain ECF flow velocity, diffusion, BBB permeability, specific and non-specific binsings were presented.

### PBPK Model Linked to IVIVE

These are mechanistic models that use the knowledge of anatomy and physiology of the brain to determine the structure of the models, combined with IVIVE strategy. The main goal is development of entirely bottom up models, where system parameters are obtained from the literature and drug specific parameters are obtained from *in silico* and *in vitro* analysis. The use of IVIVE in the development of models for brain disposition and the strategy for its application based on routinely used *in vitro* cell lines have been presented and demonstrated through applications to a number of compounds. This approach is mechanistic in natures as it enables dissection of different components of the system, therefore parameters such as *in vivo* transporter parameter can be translated from *in vitro* measuremets with the aid of appropriate scaling factors. This approach has also been successfully applied in the development of PBPK models for prediction of drugs focusing on hepatic and intestinal components, such as the permeability and transporter uptake and efflux.

Fenneteau *et al*., (55) developed a PBPK model in wild-type (WT) and mdr1a/1b (-/-) knockout (KO) mice to assess the distribution of drugs in MDR1 expressing tissues. The model focused mostly on heart and brain using tissue concentrations of domperidone. The model also takes into account apparent passive diffusion and active transport across blood tissue membranes. Important components of this model were therefore permeability-surface area product parameters and MDR1 efflux clearances. An approach for extrapolation of these parameters from *in vitro* Caco-2 monolayer experiments to *in vivo* estimates was presented. The model provided an insight into drug distribution in MDR1 expressing tissues, it also highlighted the need for quantitative knowledge on transporters from tissues and species for the potential of the approach to be realised. With model developed prior to availability of *in vivo* data, the authors also dicussed the potential use of the approach for different drugs and transporters. Yamamoto *et al*., (51,56) in the latest update of their PBPK model for brain disposition, also proposed a workflow for the use of *in silico* and *in vitro* data to inform active transport parameters across the BBB and BCSFB in the context of their model. Instead of incorporating the expression level and activity of each transporters, a parameter is used to describe the “net effect of active transporter”. This approach uses reported Kpuu values to derive the contribution of active transport to the overall process. As part of the decision tree proposed, *in vitro* kinetic parameters from endothelial cells are used when there is insufficient information for the active process from other sources. Ball *et al*., (57,58) described PBPK models for CSF and brain disposition of drugs using plasma, CSF and brain ECF concentration data from *in situ* or microdialysis in rats. The CNS model has compartments for brain vascular, CSF, brain ECF and tissue. Reference values were used for physiological parameters; blood flows and volumes. However, drug transfer across BBB and BCSFB were described using first-order bidirectional parameters with permeability-surface area constants, which were optimised using sensitivity analysis. In addition, a strategy for incorporating a bottom up IVIVE strategy in the prediction of permeability across the BBB using Caco-2 experimental data was also presented. Badhan *et al*., (59) developed PBPK models for CNS disposition of drugs in rats and mice for a number of compounds, using *in vitro* permeability data obtained from LLC-PK_1_-mdr1a cells, an entirely bottom up approach. This model was further extended to account for distribution into frontal cortex and hippocampus in addition to whole brain ECF (regional brain disgribution). The model was developed and validated in rats for carbamazepine and phenytoin and was also further extended to humans (60). Gaohua *et al*., (38) developed a 4-compartment brain model in human, nested within a whole body-PBPK model and implemented within the Simcyp Simulator (V16). The model is also based on IVIVE of transport parameters across membranes, using both system related parameters in transporter abundance and drug specific parameters in transporter function kinetics. The model was used to predict CSF concentration of paracetamol and phenytoin in humans. An adavantage of linking brain model to a whole body-PBPK model is the potentials to asses “what if” scenariois such as transporter mediated DDI, effect of age and disease conditions on drug disposition in the brain. Li *et al.,* (25,39) applied the Gaohua *et al*., (38) model in Simcyp Simulator (V16,V14) to a first-in-class drug under development, AZD1775 (39) and three cyclin D-cyclin dependent kinase 4 and 6 (CDK4/6) inhibitors (ribociclib, palbociclib, and abemaciclib) in glioblastoma patients (25). Both plasma and brain concentration were adequately predicted in patients with this bottom up approach, using *in vitro* and *in silico* data from various experiments including IVIVE of transporter kinetics data obtained from MDCKII (MDCKII-MDR1 and MDCKII–BCRP) systems. Futhermore, Verscheijden *et al.,* (61) extended Gaohua *et al*., (45) model to children by making adjustments for age dependent system and drug dependent parameters, for prediction of drug disposition in CSF of drugs that undergo passive transfer. The model was validated using four analgesics (paracetamol, ibuprofen, flurbiprofen and naproxen) and further optimised emprically to account for BBB penetration in paediatric meningitis patients using meropenem as an example.

## TRANSPORTERS IN BLOOD-BRAIN BARRIER

This section aims to give an insight into the current knowledge of the expression of transporters in the brains from a drug delivery perspective. Quantitative proteomic techniques have facilitated measurement of the protein levels of drug transporters in the brain. Several transporters are reviewed in terms of their protein expression in rat (Table III) and normal human brain tissue (Table IV) where data is available. Tables III and IV include data for the following ABC transporters: multidrug resistance protein 1 (ABCB1 or MDR1) or P-glycoprotein, the ATP-binding cassette sub-family A member 2 and 8 (ABCA2 or ABC2 and ABCA8 or KIAA0822), the multidrug resistance associated proteins (ABCC1 or MRP1, ABCC4 or MRP4, ABCC6 or MRP6) and the breast cancer resistance protein (ABCG2 or BCRP). Also, data for members of two key solute-linked carrier (SLC or SLCO) superfamilies, SLC22, SLCO21A and SLCO22A, are available, representing the organic cation transporters 1, 3 (OCT1 and OCT3), the organic cation/carnitine transporter 1 (OCTN1), the organic anion transporters (OAT1, OAT2, OAT3, and OAT7) and the organic anion transporting polypeptide (OATP1A2, OATP8, OATP2B1 and OATP1C1). There is limited information available regarding protein expression of transporters within brain tissues measured using labelled isotope standard targeted quantitative methods LC MSMS (3,4,62–69).

**Table III:** Absolute Transporter Protein Content in a Panel of Pool Rat Brain Microvessels Quantified by LC-MS/MS

	Hoshi et al., (62)^a,¥^	Gomez-Zepeda et al., (83) ^c,¥^	Al Feteisi et al., (3)^¥^	Billington et al., (64)^*^	Bao et al., (65)^a,*^	Uchida et al., (66)^b,¥^	Omori et al., (84)^b,*^
Mean ± SD							
Abcb1a/Mdr1a	19.0 ± 2.0	25.2	19.6 ± 1.35	14.1 ± 7.0 (15.8 ± 2.56)^¶^	21.9	14.3 ± 0.43 (69.5 ± 2.1)^¶^	18.0 ± 0.3
Abcc1/Mrp1	–	–	–	–	–	0.82 ± 0.12 (3.98 ± 0.61)^¶^	–
Abcc4/Mrp4	1.60 ± 0.29	–	2.16 ± 0.74	0.73 ± 0.24 (0.91 ± 0.32)^¶^	–	1.63 ± 0.03 (7.92 ± 0.16)^¶^	0.99 ± 0.03
Abca8a	–	–	–	–	–	2.80 ± 0.16 (13.6 ± 0.8)^¶^	–
Abcg2/Bcrp	4.15 ± 0.29	2.03	3.07 ± 0.46	2.98 ± 0.62 (4.07 ± 2.01)^¶^	4.59	6.24 ± 0.06 (30.4 ± 0.3)^¶^	4.56 ± 0.21
Slc2a1/Glut1	84.0 ± 4.1	–	83.5 ± 4.8	45.0 ± 25 (49 ± 12)^¶^	201.6	81.6 ± 1.35 (397 ± 7)^¶^	92.2 ± 1.6
Slc3a2/4F2hc	–	–	16.2 ± 1.90	–	–	2.27 ± 0.21 (11.1 ± 1.0)^¶^	–
Slc7a5/Lat1	3.41 ± 0.74	–	3.70 ± 0.89	–	14.7	–	0.398
Slc16a1/Mct1	11.6 ± 0.6	–	7.08 ± 1.10	–	–	8.81 ± 0.22 (42.9 ± 1.1)^¶^	10.3 ± 0.5
Slc22a8/Oat3	2.13 ± 0.49	–	1.23 ± 0.04	–	–	3.37 ± 0.12 (16.4 ± 0.6)^¶^	0.98 ± 0.05
Slc27a1/Fatp1	–	–	1.16 ± 0.62	–	–	3.34 ± 0.25 (16.3 ± 1.2)^¶^	1.61 ± 0.20
Slco1a4/Oatp1a4	–	–	1.49 ± 0.09	1.99 ± 1.03 (2.25 ± 0.90)^¶^	–	3.17 ± 0.69 (15.5 ± 3.4)^¶^	2.36
Slco1c1/Oatp1c1	–	–	–	–	–	3.18 ± 0.08 (15.5 ± 0.4)^¶^	2.40 ± 0.04

a Data are expressed as median; b Standard error of the mean (S.E.M); c averaged mean value obtained from three different days; ¥protein amount in pmol/mg of total protein; *protein amount in pmol/mg of total membrane protein; ¶ protein amount in pmol/g brain tissue

**Table IV:** Absolute Transporter Protein Content in a Panel of Individual Human Brain Microvessels Quantified by LC-MS/MS

	Uchida *et al.,* (67)^¥^	Shawahna et al., (68)^¥^	Al-Majdoub et al., (4)^¥^	Billington et al., (64)^*, ǁ^	Storelli et al., (69)^ǁ , ¶^	Uchida et al., (66)^b,¥^	Bao et al., (65)^a,*^
Mean ± SD (Fold difference)							
ABCA2/ABC2	2.86 ± 0.58 (1.9)	2.11 ± 0.78	0.08 ± 0.03 (4)	–	–	–	–
ABCA8/ABCA8	1.21 ± 0.24 (>1.75)	0.67 ± 0.23	–	–	–	0.58 ± 0.10 (1.41) (1.35 ± 0.45)^¶^	–
ABCB1/MDR1	6.06 ± 1.69 (2.1)	3.98 ± 0.88	2.58 ± 0.93 (3.2)	2.86 ± 1.75 (7.57 ± 2.31)^¶^	4.70 ± 2.0	3.91 ± 1.38 (2.01) (9.40 ± 4.90)^¶^	3.38 (5.6)
ABCC4/MRP4	0.19 ± 0.07 (2.6)	0.31 ± 0.11	–	–	–	–	–
ABCC6/MRP6	–	–	0.48 ± 0.06 (1.7)	–	–	–	–
ABCG2/BCRP	8.14 ± 2.26 (2.9)	6.15 ± 1.41	2.22 ± 0.61 (2.4)	5.64 ± 4.24 (14.2 ± 5.6)^¶^	10.8 ± 5.4	3.39 ± 0.74 (1.5) (7.92 ± 2.78)^¶^	6.21 (7.4)
SLC2A1/GLUT1	139 ± 46 (2.7)	–	21.9 ± 9.80 (9.5)	559 ± 349 (1477 ± 400)^¶^	–	32.6 ± 8.2 (1.7) (76.1 ± 28.7)^¶^	54.4 (5.0)
SLC7A5 /LAT1	0.43 ± 0.09 (>2.03)	0.80 ± 0.25	0.65 ± 0.17 (2.5)	–	–	–	3.42 (6.3)
SLC16A1/MCT1	2.27 ± 0.85 (3.2)	1.46 ± 0.39	5.37 ± 3.73 (13.4)	–	–	1.59 ± 1.54 (5.8) (2.90 ± 1.44)^¶^	5.70 (19.1)
SLC16A2/MCT8	–	1.31 ± 0.37	–	–	–	2.34 ± 0.08 (1.06) (5.71 ± 2.44)^¶^	–
SLC19A1/RFC	0.76 ± 0.04 (>1.42)	0.55 ± 0.18	0.18	–	–	–	–
SLC22A1/OCT1	–	–	0.56 ± 0.07 (1.5)	–	–	–	–
SLC22A3/OCT3	–	–	0.62 ± 0.08 (1.5)	–	–	–	–
SLC22A4/OCTN1	–	–	0.04 ± 0.01 (2.4)	–	–	–	–
SLC22A6 /OAT1	–	–	0.48 ± 0.11 (2.1)	–	–	–	–
SLC22A7 /OAT2	–	–	7.90 ± 3.80 (6.5)	–	–	–	–
SLC22A8 /OAT3	–	–	0.27 ± 0.03 (1.5)	–	–	–	–
SLC22A9/OAT7	–	–	0.51 ± 0.10 (2.0)	–	–	–	–
SLC29A1 /ENT1	0.57 ± 0.13 (1.85)	0.86 ± 0.13	0.27 ± 0.10 (3.6)	0.64 ± 0.43 (1.86 ± 0.85)^¶^	–	–	–
SLC44A1/CTL1	–	–	0.88 ± 0.62 (7.9)	–	–	1.92 ± 0.34 (1.3) (4.85 ± 2.70)^¶^	–
SLC44A2/CTL2	–	–	1.14 ± 0.75 (10.8)	–	–	5.80 ± 1.47 (1.5) (13.2 ± 3.3)^¶^	–
SLCO1A2/OATP1A2	–	–	0.54 ± 0.10 (1.9)	–	–	–	–
SLCO1B3/OATP8	–	–	0.46 ± 0.15 (5.5)	–	–	–	–
SLCO2B1/OATP2B1	–	–	0.40 ± 0.04	0.37 ± 0.26 (0.93 ± 0.38)^¶^	0.64 ± 0.43	–	–
SLCO1C1 /OATP1C1	–	–	0.27 ± 0.03 (1.4)	–	–	–	–

^a^Data are expressed as median; ^b^Standard error of the mean (S.E.M); ^¥^protein amount in pmol/mg of total protein; ^*^protein amount in pmol/mg of total membrane protein; ^ǁ^BA39 (parietal lobe, all other studies either cortex or frontal cortex); ^¶^protein amount in pmol/g brain tissue

Reported abundance data for rats and humans were collated and compared. Overall, expression of Mdr1a or MDR1 was the highest in rats (mean, 18.4 pmol/mg of total protein), while bcrp or BCRP was the highest in human (mean, 4.26 pmol/mg of total protein), and in both species SLC family transporter (Glut1/GLUT1) were the most abundant (mean in rat, 77.3 pmol/mg of total protein; mean in human 188 pmol/mg of total protein, respectively). Among the most highly expressed transporters, more monocarboxylate transporter 1 (Mct1/MCT1) was present in the rats compared to humans (mean, 9.4 versus 2.7 pmol/mg total protein, respectively). This transporter allows the entry of lactate and ketone bodies into the brain. More amino acid transporter (Lat1/LAT1) was expressed in the rats (mean, 2.6 pmol/mg protein) compared to the humans (mean, 0.63 pmol/mg total protein). Most rat abundance data from the individual studies were comparable and within 2-fold of difference except for one study (65), where the transporter abundance values were reported as median (not mean) and not included in calculating the weighted mean from all studies, mostly indicating differences between these studies. Unsurprisingly, protein abundance data for mdr1a or MDR1 and bcrp or BCRP were reported in all collected studies.

**Fig. 2:**
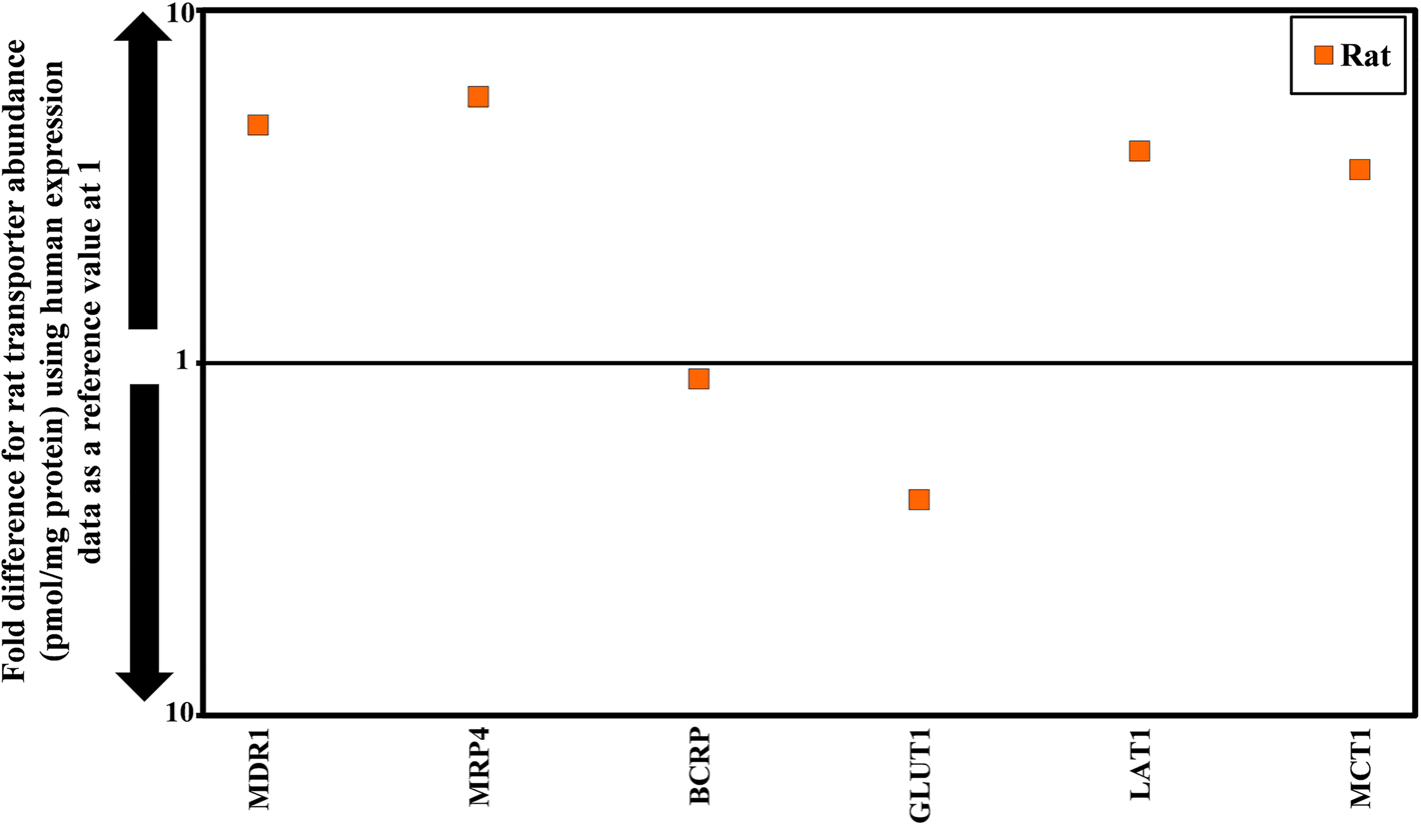
Comparison of fold difference in the expression of 6 transporters in rat brain microvessels relative to the human abundance data. Abundance data from two studies: Bao *et al*., (65) and Storelli *et al*., (69) was excluded from comparison where data reported as median in Bao *et al*., (65) and in g/brain tissue in Storelli *et al*., (69)

Clearly, differences in transporters expression exist between species. For instance, Mdr1a/MDR1 expression was reported to be higher in rats than in humans (>10-fold difference, Fig. 2) and the Mann-Whitney test showed significant differences (*p* = 0.0043). Bcrp/BCRP was nevertheless shown to be similar between rats and humans (*p* = 0.25, Fig. 2). Mdr1 protein expression in rat brains within and in between studies was not very variable (mean 18.4 pmol/mg of total protein, % CV 22). Protein expression levels of MDR1 in the human brains were reported not to be statistically different between all studies (<2.5-fold difference). The Mrp/MRP expression are reported in rat (Mrps 1, 4) and human brains (MRP4, 6). Mrp1 and MRP6 were reported in only one study; hence, a comparison was not possible. On the other hand, Mrp4/MRP4, are expressed in both human and rat and the abundance difference was 6-fold lower in human than rat (Fig. 2) and within the 3-fold difference observed between rat studies. Non-parametric statistics test (Mann-Whitney test, *p* =0.9) showed no differences (*p* =0.9) for Glut1/GLUT1 between rat and human brains (Fig. 2). Between studies however, rat expression data revealed for Glut1 expression a 5-fold difference, while, human expression data showed an over 20-fold difference. Those conflicting results can likely be explained by differences in tissue conditions (i.e., fresh compared to frozen), storage conditions and due to different methodology, that have been used (70) but perhaps more likely to the varying clinical background and post-mortem status of the brain tissues. The expression levels of other transporters such as Lat1, Mct1, Oat3, Oatp1a4, 4f2hc, Fatp1, ABC2, ABCA8, RFC, OATP2B1, MRP4, MCT8, CTL1, and CTL2 were reported in either two or three studies. The reports on most transporters’ expression are comparable between studies; except for expression of 4f2hc, Fatp1, ABC2, CTL2, RFC ranging from 3- and 35.7-fold difference. The expression pattern of Mrp1, Oatp1C1, MRP6, OCT1, OCT3, OCTN1, OAT1, OAT2, OAT3, OAT7, and OATP1C1 is still unclear. These transporters were quantified in only one study (4,66) but there is convincing mRNA evidence for expression of these transporters at BBB (70).

## DISCUSSION AND CONCLUSIONS

“Science is built of facts as a house is built of stones but an accumulation of facts is no more science than a pile of stones is a house”

Henri Poincare, 1828–1892

More complex PBPK brain models have appeared in the literature recently, reflecting the physiology/anatomy/biology of CNS components. The level of added complexity has been mirroring the intended sophisticated use of the models. The models have helped to turn the sparse and silo pieces of information into an integrated knowledge of the drug disposition in brain.

**Fig. 3:**
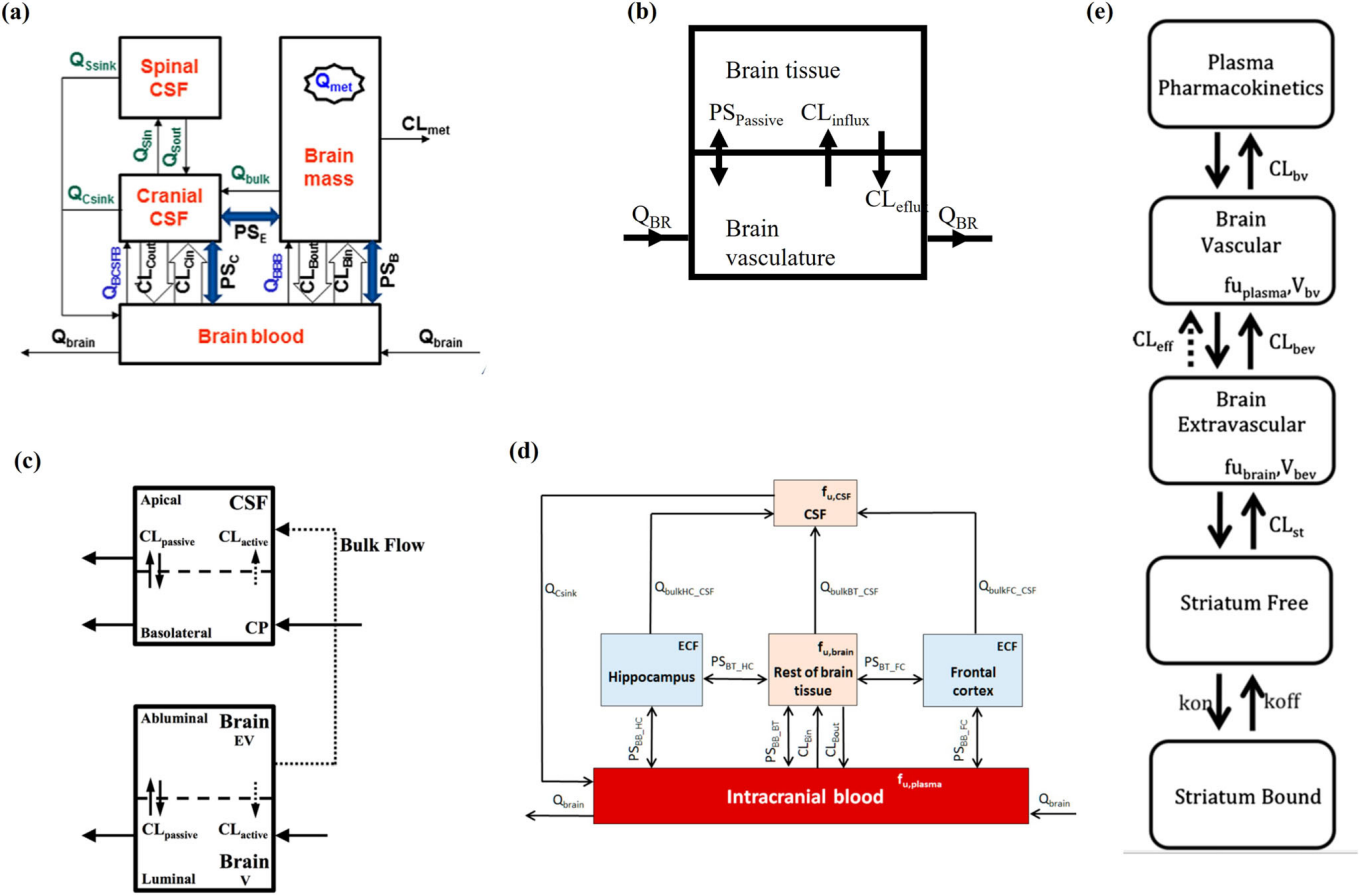
Schematic representation of the CNS components of the IVIVE models that have considered absolute transporter abundance in their implementation: **a** Li *et al.,* (25,39) (implemnted in Simcyp Simulator Gaohua *et al*., (38)), **b** Ball *et al*., (57), **c** Badhan *et al*., (59), **d** Zakaria *et al.*, (60), **e** Johnson *et al*., (71). (All figures are reproduced with pernission)

Since direct measurement of human CNS concentrations is associated with many obstacles, it is important to consider the use of these models. Nonetheless, obtaining appropriate model parameters in order to understand the central kinetics of drugs in humans is a challenge considering various diverse methods used for parameters that are used to describe such models. The IVIVE of permeability by scaling with absolute transporter abundance to the PBPK model is a fundamental aspect in translational abilities to humans whether from *in vitro* experiments or from non-clinical data. Of the IVIVE PBPK models that we could identify, only five model have considered absolute transporter abundance (39,57,59,60,71). Schematic representation of the CNS components of these models are shown in Fig. 3, their properties are summarised in “PBPK model linked to IVIVE” section above and detailed description can be found in the references. With the increasing number of reports on absolute transporter abundance in recent years, this leaves an opportunity to refine these IVIVE-PBPK models so they can be the next generation of tools for a more successful CNS drug development.

Model-informed drug development (MIDD) is no longer just an aspirational idea. There are plenty of evidence to suggest this has become an integral part of modern drug development by leading pharmaceutical companies (72,73) and all the signs are in place from the top regulatory agencies that MIDD is seen not only acceptable approach in many cases but also an encouraged path in certain circumstances (74–77). Therefore, the pharmaceutical industry is arriving at a conjuncture where ability to incorporate the MIDD paradigm into widespread practice is not just an internal scientific decision; it is rather a management strategy issue (78). As with many other management tasks this requires careful assessment of implementation (if MIDD nucleus is not in place) and scaling regarding many other elements (personnel, tools, environment and processes). Traditionally, modelling and simulation (M&S) have been performed by specialised teams who create bespoke models for each case and have reservations about letting modelling be done by the greater mass of scientists engaged in various stages of drug development. This approach is proven to be too restrictive in the current MIDD environment. As MIDD enters mainstream use during drug development by many pharmaceutical companies, community assessment of various models applied to a certain problem and settling on some selected models that can be used repeatedly by a mass of users with assurance on reproducibly of results become inevitable. This is distinct from the somewhat academic research-oriented use (78).

The current review of PBPK-IVIVE link models suggest that these models are coming to a degree of maturity that they warrant wider applications to real world drug development issues by a wider community of the drug developers that early adopters who take positions in risky frontiers of diverting from the norm. However, there are also gaps in many aspects that need to be addressed, and subsequently get integrated into models rather than remaining as mere facts.

Combining *in silico* and *in vitro* data with preclinical *in vivo* animal experiments and clinical studies in humans provides an opportunity for these IVIVE-PBPK models to be verified through reverse translation (79). This provides robust quality assured models for the next generation of tools used in successful CNS drug development, particularly with added connections to quantitative system pharmacology model (80).

## Declarations

### Disclaimer

Views expressed in this article are personal opinion of the authors themselves, formed by their scientific judgment, and do not reflect policies, view or required practices of affiliated organizations.
